# Needs assessment for interprofessional education module on prevention and early detection of oral cancer among dental interns: a cross- sectional survey

**DOI:** 10.1186/s12903-024-05123-7

**Published:** 2024-11-07

**Authors:** Nanditha Sujir, Junaid Ahmed, Anand Ramakrishna, Ciraj Ali Mohammed, Bhaskaran Unnikrishnan, John HV Gilbert

**Affiliations:** 1 Department of Oral Medicine and Radiology, Manipal College of Dental Sciences Mangalore, Manipal Academy of Higher Education, Manipal, Karnataka 576104 India; 2 Department of Respiratory Medicine, Kasturba Medical College Mangalore, Manipal Academy of Higher Education, Manipal, Karnataka 576104 India; 3 Department of Medical Education, Kasturba Medical College Mangalore, Manipal Academy of Higher Education, Manipal, Karnataka 576104 India; 4 Medical Education, College of Medicine and Health Sciences, National University of Science and Technology, Muscat, Oman; 5 Department of Community Medicine, Kasturba Medical College Mangalore, Manipal Academy of Higher Education, Manipal, Karnataka 576104 India; 6 University of British Columbia, Vancouver, Canada; 7 WHO Collaborating Centre on Health Workforce Planning and Research, Dalhousie University, Halifax, Canada; 8 University of Technology, Sydney, Australia; 9 Interprofessional Education and Care, Manipal Academy of Higher Education, Manipal, 576104 India

**Keywords:** Interprofessional Education, Oral Cancer, Prevention, Screening, Interprofessional learning

## Abstract

**Background:**

The challenges associated with ensuring widespread system changes to enable early diagnosis and prevention of oral cancer could benefit from interprofessional practice. A needs assessment study was conducted to inform the Interprofessional Education and Collaborative Practice (IPECP) course related to oral cancer. The primary objectives of this study were 1) to establish a tool assess the knowledge attitude and practice (KAP) related to prevention and early detection of oral cancer of health professional students, and 2) to assess the same KAP of pre-licensure dental students. Additional objectives were to consider the possibility that dental students would demonstrate good scores related to early detection and prevention of oral cancer thus indicating their readiness for interprofessional learning and collaborative practice.

**Methods:**

Two questionnaires were utilized for this study which included 1) Readiness for interprofessional learning was assessed using the pre- validated tool of Readiness for Interprofessional Learning Scale (RIPLS) 2) A questionnaire to assess the KAP related to early diagnosis and prevention of oral cancer which was developed, validated, and evaluated. Statistical analysis includes, descriptive statistics, Mann–Whitney U test, Ordered logistic regression and Probit analysis. *p* value was set at < 0.05.

**Results:**

A total of 130 dental students (74.6% female) were included in the study. Mean scores related to KAP were 15.96 ± 1.394, 4.70 + 1.146, 7.02 ± 1.019 respectively. The mean score of RIPLS was 73.15 ± 15.961. The probability of overall samples to have good RIPLS scores was around 0.68 to 0.76 (Male 0.68—0.82 & Female 0.68 -0.74). The percentage of students having good *knowledge score* was 93.8%, good *attitude score* was around 54.6% and good *practice score* was around 90%.

**Conclusion:**

Knowledge and practice related to prevention and early detection of oral cancer were scored highly. Attitude scores were lower in a relatively higher proportion of participants and needed to be addressed in the curriculum. RIPLS score indicates a positive attitude towards interprofessional learning.

**Supplementary Information:**

The online version contains supplementary material available at 10.1186/s12903-024-05123-7.

## Background

Oral cancer poses a significant burden on health outcomes, being the 18th most common cancer worldwide [1]. According to reports by World Health Organization (WHO), the South-East Asian region has the highest incidence rate (8.0) and mortality rate (4.5) for oral cancer. The yearly incidence of lip and oral cavity cancer was more than 100,000 in India [2]. In India, most patients present in an advanced stage of the disease with extremely poor prognosis. Delay in diagnosis of oral cancer further escalates the socioeconomic burden imposed by the disease. Thus, measures for early detection and prevention of oral cancer are critical to assure favorable outcomes. A recent study concluded that conventional screening technique for high-risk population is the most cost-effective method for population-based surveillance for oral cancer [3]. Other modalities for screening of oral cancer such as toluidine blue staining, oral cytology, and light-based detection are available in specific clinical settings and are not readily available in routine clinical practice. This makes conventional screening practices the most feasible method with scope for wide application for early diagnosis of oral cancer [3]. Prevention of oral cancer largely focuses on tackling the most common causative agents, namely tobacco and alcohol. In India, the addition of betel quid chewing is another important etiology that needs to be considered. Routine practice of educating patients and providing cessation services is an integral aspect of prevention of oral cancer. Additionally, recognizing potentially malignant disorders and managing them effectively contributes significantly to preventive efforts [4].

Apart from widespread community screening efforts that have been successful [5, 6], dental practitioners are key health care professionals embedded within the community, who should contribute to the efforts of early detection and prevention of oral cancer [7, 8]. Thus, it’s incumbent that the future dental practitioners are provided with the necessary competencies to contribute to oral cancer prevention and early diagnosis during their training period. As this aspect forms a necessary concern in the efforts to promote early diagnosis and prevention of oral cancer, the role of other health professionals cannot be ignored [8–10]. Poor public awareness related to oral cancer coupled with lack of awareness related to the scope of work of dental practitioners can lead patients to seek care for oral symptoms from a primary care physician [8]. Other health professionals, who undergo limited training in the management of oral disease, particularly medical, nursing and speech language pathologist are placed at potential vantage points in the healthcare system and could potentially contribute to oral cancer prevention and early detection.

To improve the outcomes related to oral cancer, the overarching aim would be to develop a health care system that caters to identification of at-risk population, which provides timely screening, intervention and long-term follow-up of individuals. It calls for improved awareness and change in behaviors among individuals and/or community members at large, as well as health care professionals. The complexity of the situation would demand interprofessional collaboration that provides for individual/community centered care. The “World Health Organization (WHO)” has recognized “interprofessional education (IPE)” as critical to addressing the global health professional workforce crisis [11]. IPE provides a framework for integration of non-dental workforce in endeavors to promote oral health [12]. Until the students are given the opportunity to learn together in an interprofessional group, expecting collaboration between various professions in a work environment would be a challenge [11, 12]. Interprofessional education (IPE) has been developed to foster competencies for collaborative care among students, such as patient centered team-based care, cognizance of roles and responsibilities of all involved health professionals, good communication, ability to manage conflicts, interprofessional ethics and values etc. (CIHC, 2010; IPEC 2016) [13, 14]. IPE helps to create an environment for interprofessional students to develop mutual trust, and respect among professionals enabling sustainable collaborative health care delivery [15]. Considering these factors, our aim is to develop an interprofessional education module for prevention and early detection of oral cancer for health professional students. Contributing to this endeavor we sought to establish a baseline related to knowledge, attitude and practice (KAP) for prevention and early detection of oral cancer among health professional students and their readiness for interprofessional learning. Thus, the primary objectives of the present study was 1) to develop a tool to assess the KAP related to prevention and early detection of oral cancer of different health professionals, and 2) to assess the same among dental students. An additional objective was to explore the probability of these students to have good scores related to early detection and prevention of oral cancer and readiness for interprofessional learning.

## Material and methods

### Research design

The cross-sectional study was conducted after obtaining clearance from the Institutional Ethics Committee from Manipal College of Dental Sciences Mangalore (Protocol No: 22099). This report was written in consideration of the Strengthening the Reporting of Observational studies in Epidemiology (STROBE) statement (Supplementary file 1) [16]. Informed consent was obtained prior to recruiting participants.

### Survey tool

Two questionnaires were utilized for this study. Readiness for interprofessional learning was assessed using the pre- validated tool of Readiness for Interprofessional Learning Scale (RIPLS) questionnaire [17]. The questionnaire to assess the KAP related to oral cancer prevention and early detection was developed by modifying and adapting the questionnaire developed by Shubayr et al.,[18]. Considering the risk factors of oral cancer specific to Indian demographics, early diagnosis and prevention of oral cancer, and the target population, several items were added, deleted or rephrased to formulate a 44-item questionnaire. After expert review (Four experts in the field of Oral medicine and Oral pathology) 8 items were excluded considering a 36-item questionnaire was finalized. The new items added were the questions, “Are you aware of potentially malignant diseases of the oral cavity?”, “Can oral cancer occur in young individuals?” “Is early oral cancer always associated with symptoms?”, Should annual oral cancer examinations be provided for individuals with gutka chewing habit?.” The question “Do you take biopsy in suspected oral lesions was rephrased to “Do you advise biopsy in suspected oral lesions?” considering the target population. The question “Patients’ with suspected oral cancerous lesions should be referred to a specialist” was rephrased to “Do you refer patients with suspected oral cancer to a specialist?” for uniformity. Additionally, several questions related to tobacco and alcohol cessation were added considering its importance in prevention of oral cancer e. g “Are you aware of the 5 A model used for tobacco cessation intervention?” The first part of the questionnaire includes information age, gender, profession etc. Eighteen statements (item no. 1 to 17 & 23) were used to assess knowledge, six statements (item no.18 to 21,26, 33) to assess attitude and eight statements (item no. 22,24,25,27 to 31) to assess practice. The response options were yes and no, and correct responses were given a score of 1. Thus, knowledge domain had a maximum of 18 points, attitudes 6 points and practice 8 points. Average score was computed, and good or poor outcomes were defined based on the results. The last 3 items on the questionnaire were to assess the willingness of students to learn about early diagnosis of oral cancer and its prevention.

### Content validity

Content validity was assessed by distributing this modified questionnaire among a panel of 8 expert members belonging to the specialty of oral medicine, oral pathology, community medicine, public health dentistry, nursing, and speech pathology each with more than 10 years of academic/clinical experience. The rating was based on relevance, clarity, simplicity, and ambiguity. The criteria suggested by Ayre and Scally (2014) [19] was utilized to check the content validity ratio (CVR) [CVR = (Ne—N/2)/(N/2), where: Ne: is the number of panelists who indicate an item is "essential" N: is the total number of panelists] of each item included. All items included had a content validity ratio of 1.

### Reliability

Reliability of the questionnaire was examined by the test–retest method among 50 participants; 10 students each from different professions of medicine, dentistry, speech language pathology and nursing. The questionnaire was administered twice with two weeks intervals in between. Results from the two responses were compared using Pearson’s correlation coefficient (Pearson’s r) as a reliability test. Additionally, Reliability statistics for different domains of questionnaire as well as the overall questionnaire were evaluated using Cronbach’s Alpha.

### Study participants

The sample size was calculated using fo ${z}_{POwER}$ rmula for multiple linear regression. The values for the formula were obtained by pilot testing the questionnaire on 50 interprofessional participants. Sample size formula used was N= $\frac{{\left({z}_{1-\alpha }+{z}_{POwER}\right)}^{2}{\sigma }^{2}}{{\Delta }^{2}}$ $+\left(L+K\right)$ (where, Z_1-α_ = Z-score for the desired confidence level Z_POWER_ = Z-score corresponding to the desired power, σ^2^ = error variance expected effect size (difference in R2), L = number of predictors (independent variables) K = number of covariates or additional terms) with values of alpha error of 0.05, delta as 0.3889, to detect R^2^ of 0.2800, with 90% power sample was estimated to be 108. Adjusting for 20% non-response the final sample size was estimated to be 130 students. Inclusion Criteria was students in the final year of their academic program (interns) enrolled in undergraduate dental (BDS) students. Exclusion Criteria: Students who do not provide consent to participate in the study and students who participated in the pilot study. The self-administered questionnaires were distributed among participants through a link for Microsoft Forms through email or WhatsApp messenger service. A non-probability, convenience sampling technique was followed. The questionnaire was distributed in two dental colleges in south Indian cities of Mangalore and Manipal. The permission of the dean of the college was sought to distribute the questionnaire. The questionnaire was distributed through WhatsApp to students in Mangalore and by email to the students in Manipal.

Statistical analysis was performed using STATA statistical software (Stata Corp, College Station, Texas, USA, Version 17). The descriptive statistics pertaining to demographic variables such as age and gender were represented using mean, standard deviation, frequency and percentage distribution. The descriptive statistics for overall sample, male and female with regards to overall knowledge, attitude, practice, and RIPLS score were represented using mean, standard deviation, standard error of mean, minimum, maximum and 95% confidence interval. Intergroup comparison between male and female for overall knowledge, attitude, practice and RIPLS score was performed using Mann–Whitney U test. Frequency and percentage distribution was also computed for different categories of knowledge, attitude, practice RIPLS scores. Students with scores 0–13 in knowledge domain were graded as having poor knowledge and those having scores greater than or equal to 14 were graded as having good knowledge. Students with scores 0–4 in attitude domain were graded as having unfavourable attitude and those having scores greater than or equal to 5 were graded as having favourable attitude. Students with scores 0–5 in practice domain were graded as having poor practice scores and those having scores greater than or equal to 6 were graded as having good practice scores. Students with scores 0–69 in RIPLS domain were graded as having poor RIPLS scores and those having scores greater than or equal to 70 were graded as having good RIPLS scores. The cut of score was decided based on quartile distribution with probability transformation. Ordered logistic regression was performed for RIPLS scores to determine the influence of factors such as age, gender, knowledge, attitude, practice categories. Probit analysis was performed to determine the probability of getting good RIPLS scores for different categories of knowledge, attitude and practice scores as well as gender.

## Results

The questionnaire was distributed to 150 students of which, 130 responded (86.7% response rate), of which 97 (74.6%) were female. The mean age of the study population was 23.21 ± 1**,** Reliability statistics revealed that the knowledge domain (Cronbach’s Alpha = 0.953), attitude domain (Cronbach’s Alpha = 0.987), practice domain (Cronbach’s Alpha = 0.979), overall KAP questionnaire (Cronbach’s Alpha = 0.969) and RIPLS score (Cronbach’s Alpha = 0.994) had excellent internal consistency. The descriptive statistics for overall sample, with regards to overall knowledge, attitude, practice, and RIPLS score were represented using mean, standard deviation, standard error of mean, minimum, maximum and 95% confidence interval **(**Table 1). Descriptive statistics for male and female with intergroup comparison by sex reveal that there was no statistically significant difference between the compared groups for overall knowledge score (*p* = 0.47), overall attitude score (*p* = 0.817), overall practice score (*p* = 0.887) and overall RIPLS score (*p* = 0.554) (Table 2).

**Table 1:** Descriptive statistics for overall sample (Knowledge score, attitude score, practice score and RIPLS score)

Variables	N	Mean	Std. Deviation	Std. Error	95% Confidence Interval for Mean		Minimum	Maximum
					Lower Bound	Upper Bound		
Overall knowledge score	130	15.96	1.394	.122	15.72	16.20	12	18
Overall attitude score	130	4.70	1.146	.100	4.50	4.90	2	6
Overall practice score	130	7.02	1.019	.089	6.84	7.19	4	8
RIPLS score	130	73.15	15.961	1.400	70.38	75.92	22	95

**Table 2:** Intergroup comparison performed using Mann–Whitney U test

Study variables		N	Mean	Std. Deviation	Std. Error	95% Confidence Interval for Mean		Minimum	Maximum	Test statistic	*p* value
						Lower Bound	Upper Bound				
Overall knowledge score	Male	33	15.79	1.536	0.267	15.24	16.33	12	18	1731.5	0.470
	Female	97	16.02	1.346	0.137	15.75	16.29	13	18		
	Total	130	15.96	1.394	0.122	15.72	16.20	12	18		
Overall attitude score	Male	33	4.70	1.334	0.232	4.22	5.17	2	6	1559	0.817
	Female	97	4.70	1.082	0.110	4.48	4.92	2	6		
	Total	130	4.70	1.146	0.100	4.50	4.90	2	6		
Overall practice score	Male	33	6.97	1.185	0.206	6.55	7.39	4	8	1575.5	0.887
	Female	97	7.03	0.962	0.098	6.84	7.22	4	8		
	Total	130	7.02	1.019	0.089	6.84	7.19	4	8		
RIPLS score	Male	33	72.24	15.002	2.612	66.92	77.56	32	95	1711	0.554
	Female	97	73.46	16.338	1.659	70.17	76.76	22	95		
	Total	130	73.15	15.961	1.400	70.38	75.92	22	95		

The percentage of students having good *knowledge score* was 93.8% **(**Table 3). The percentage of students having favourable *attitude score* was around 54.6% (Table 3). The percentage of students having good *practice score* was around 90% (Table 3). The percentage of students having good *RIPLS score* was around 69.2% (Table 3).

**Table 3:** Frequency and percentage distribution of students (for knowledge, attitude, practice, RIPLS scores)

Scores	Frequency	Percentage
Knowledge		
(1) Poor (0–13)	8	6.2%
(2) Good (≥ 14)	122	93.8%
Attitude		
(1) Unfavourable (0–4)	59	45.4%
(2) Favourable (≥ 5)	71	54.6%
Practice		
(1) Poor (0–5)	13	10%
(2) Good (≥ 6)	117	90%
RIPLS		
(1) Poor (0–69)	40	30.8%
(2) Good (≥ 70)	90	69.2%

Ordered logistic regression for RIPLS score revealed it was not significantly influenced by any factors such as age, gender, categories of knowledge, attitude and practice scores **(**Table 4**)** but the odds of having good RIPLS scores was around 3.08 for students with good knowledge, 2.84 for students with good practice scores and 1.49 for students with favourable attitude in comparison with students having poor knowledge, poor practice scores and unfavourable attitude scores respectively **(**Table 4).

**Table 4:** Ordered logistic regression of RIPLS scores

RIPLS	Odds Ratio	Standard Error	Z	*P* >|z|	95% Confidence interval	
Age	0.963	0.200	-0.18	0.85	0.64	1.44
Female*	0.891	0.422	-0.24	0.809	0.35	2.25
Good Knowledge Scores	3.08	2.44	1.42	0.154	0.65	14.54
Good Attitude Scores	1.49	0.627	0.96	0.339	0.654	14.54
Good Practice Scores	2.83	1.96	1.50	0.132	0.729	11.05
Constant	0.43	4.86			-9.11	9.97

*Abbreviation*: *RIPLS* Readiness for interprofessional learning

^*^Male was taken as a baseline

**Fig. 1:**
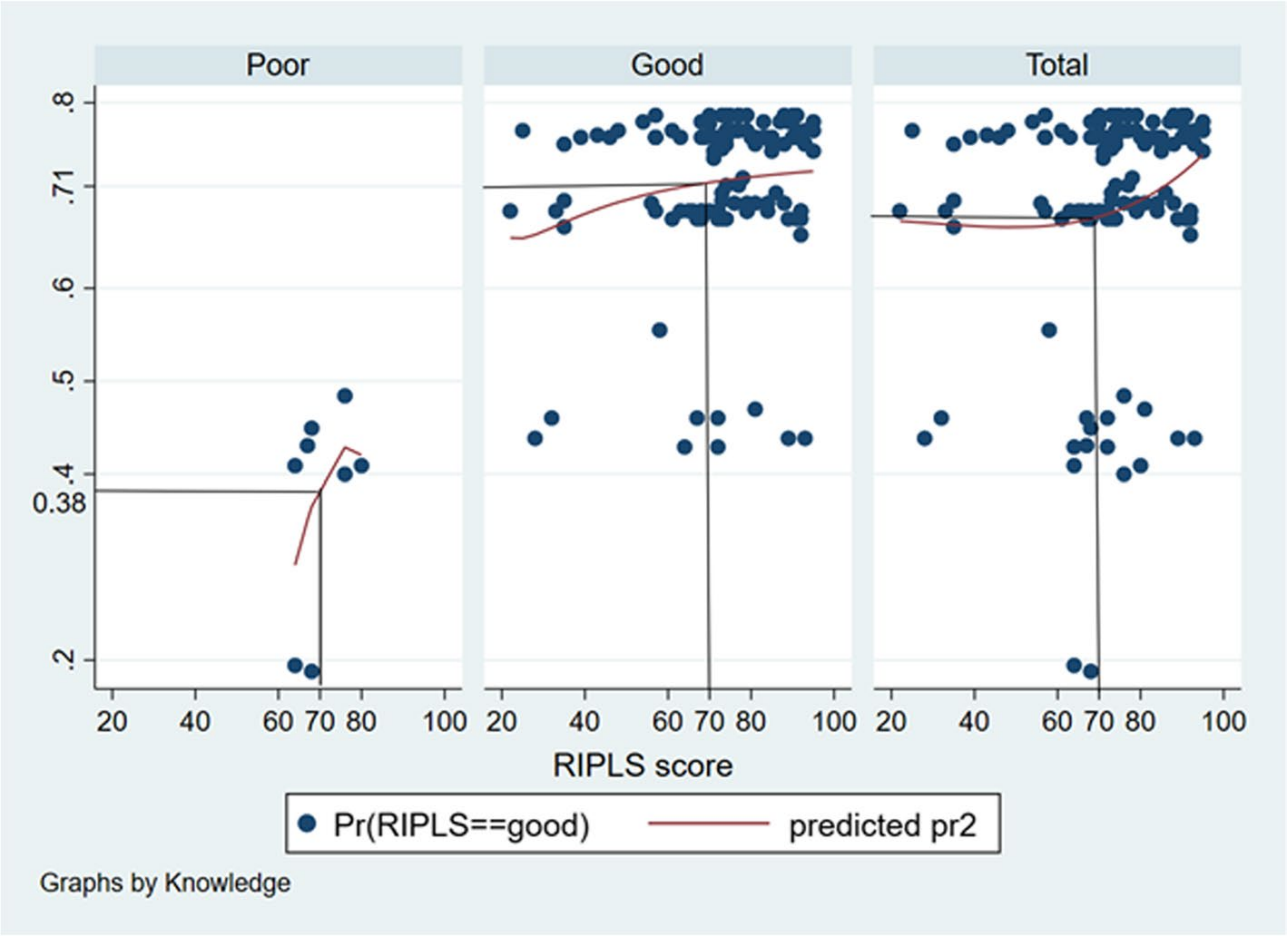
Probit graph showing the probability of good RIPLS score in relation to knowledge score

The probability of students having poor knowledge scores to possess good RIPLS score was around 0.38 to 0.46 (Fig. 1). The probability of students having good knowledge scores to possess good RIPLS score was around 0.71 to 0.73 (Fig. 1). The probability of overall samples to have good RIPLS scores was around 0.68 to 0.76 (Fig. 1).

**Fig. 2:**
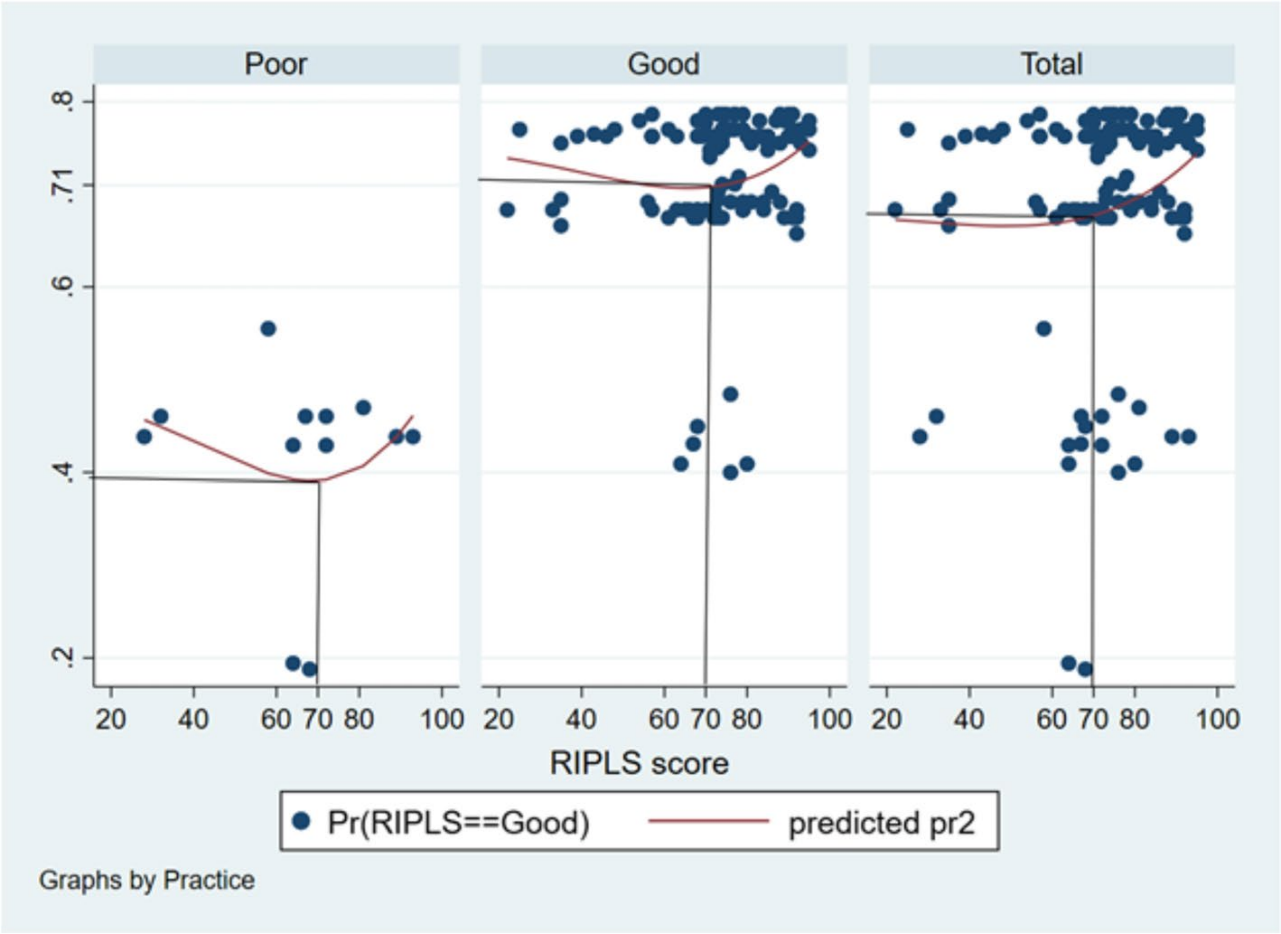
Probit graph showing the probability of good RIPLS score in relation to practice score

The probability of students having poor practice scores to possess good RIPLS score was around 0.39 to 0.49 (Fig. 2). The probability of students having good practice scores to possess good RIPLS score was around 0.71 to 0.76 (Fig. 2). The probability of overall samples to have good RIPLS scores was around 0.68 to 0.76 (Fig. 2).

**Fig. 3:**
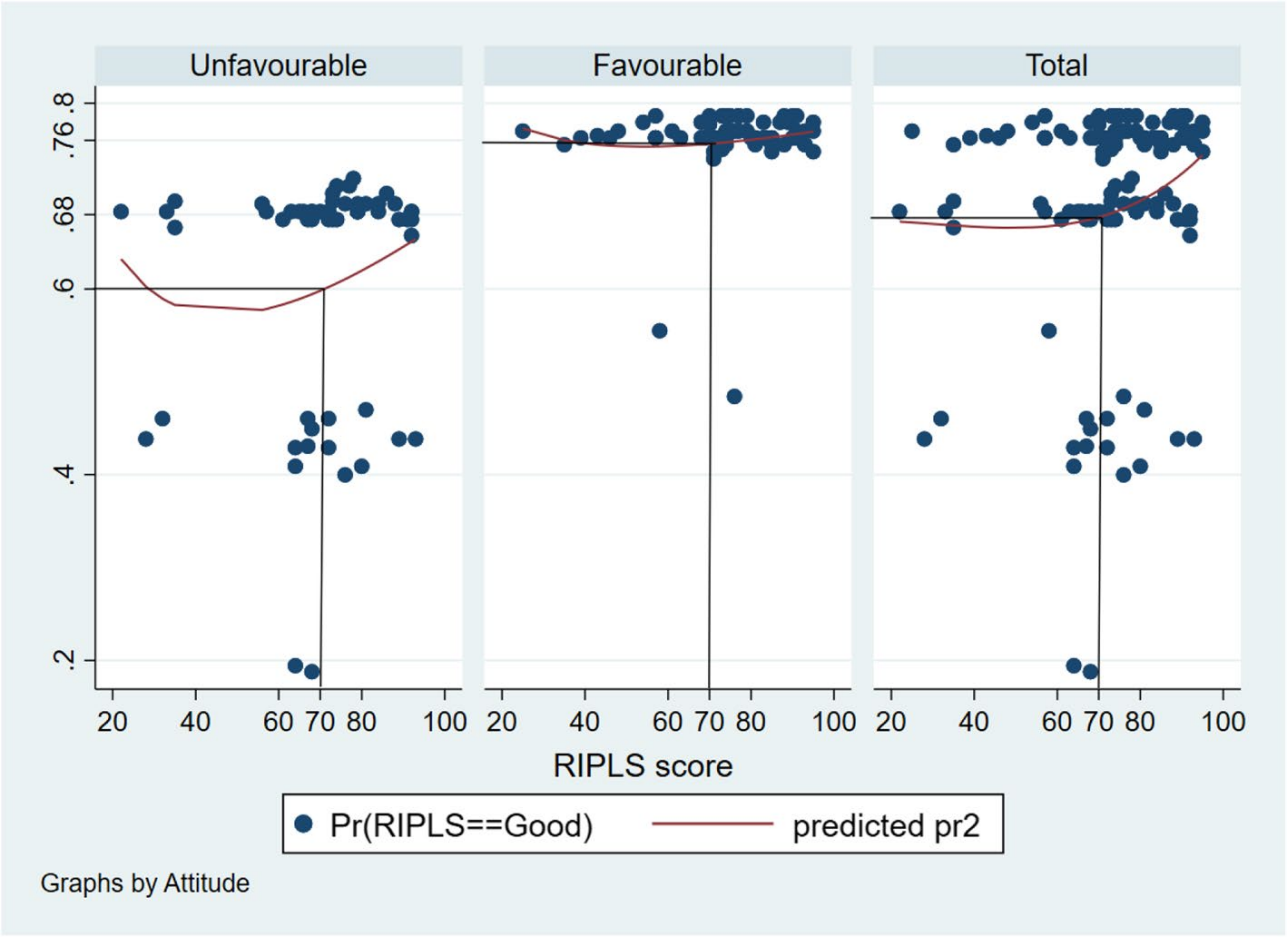
Probit graph showing the probability of good RIPLS score in relation to attitude score

The probability of students having unfavourable attitude scores to possess good RIPLS score was around 0.6 to 0.65 (Fig. 3). The probability of students having favourable attitude scores to possess good RIPLS score was around 0.76 to 0.78 (Fig. 3). The probability of overall samples to have good RIPLS scores was around 0.68 to 0.76 (Fig. 3).

Also, Majority of the participants were willing to learn more about oral cancer prevention and early detection (100%) and about tobacco and alcohol cessation education (99%). 52% of the participants felt that they did not receive training in health communications.

**Fig. 4:**
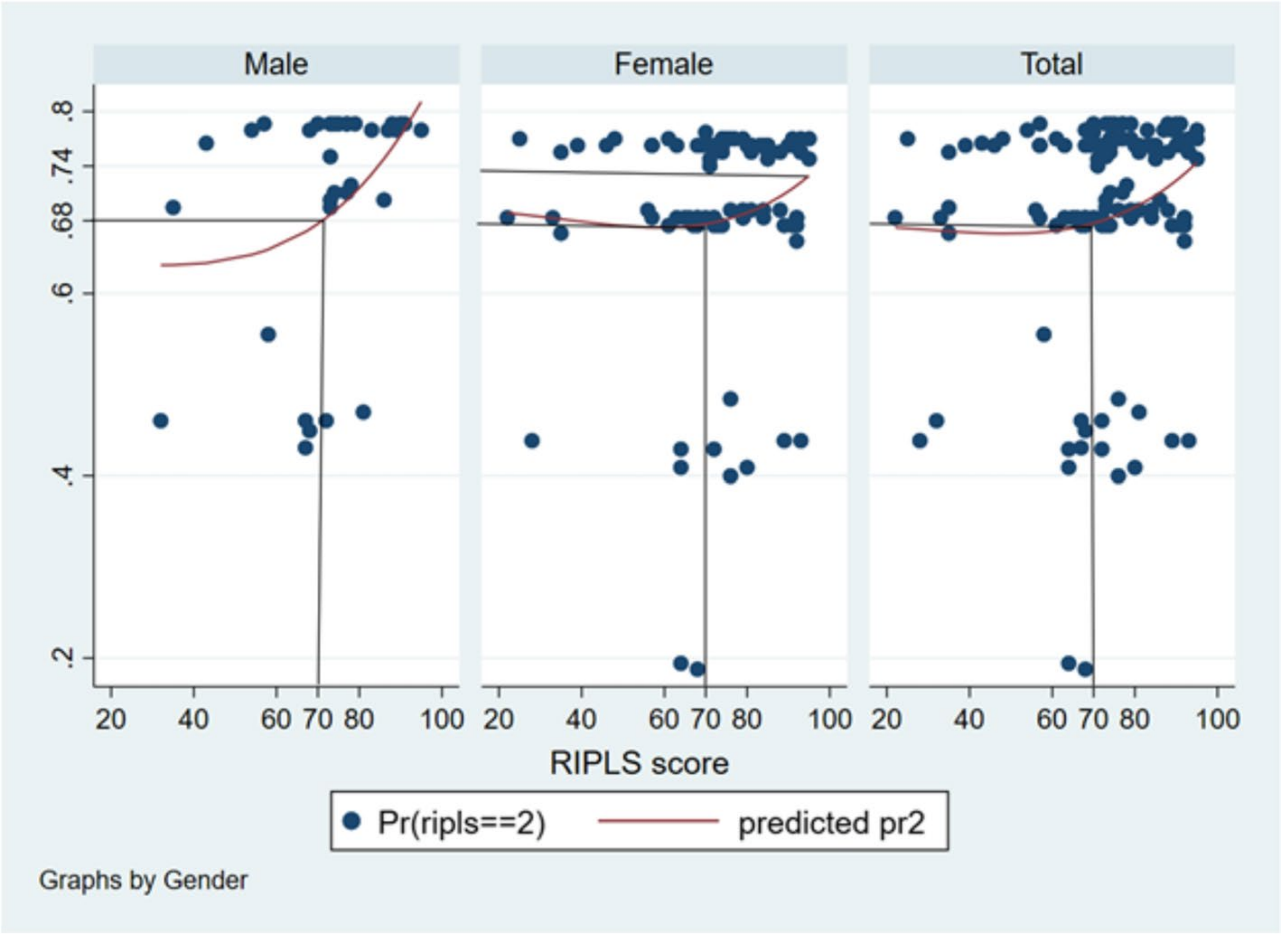
Probit graph showing the probability of good RIPLS score in relation to gender

The probability of males having good RIPLS scores was around 0.68 to 0.82. The probability of females having good RIPLS scores was around 0.68 to 0.74 (Fig. 4).

## Discussion

The objectives of this study were to establish a tool to assess the knowledge attitude and practice (KAP) related to prevention and early detection of oral cancer of health professionals, and to assess the same KAP of pre-licensure dental students. We also explored the possibility that these students would demonstrate good scores related to early detection and prevention of oral cancer thus indicating their readiness for interprofessional collaborative learning.

In this section we present the results of the needs assessment that will contribute to the development of an interprofessional education module for health professional students related to prevention and early detection of oral cancer. The tools selected for need assessment were chosen giving consideration to the academic environment, intended IPE module and cohort of interprofessional students that would participate in the education module. The reliability of the survey tools used for this study is a good indicator that the results of the survey contribute to the objective of the study. This paper focuses on the results related to KAP of dental interns related to prevention and early detection of oral cancer, as well as the readiness for interprofessional education. The results related to dental interns is presented separately considering the speciality focused on oral disease management. Diseases related to oral cavity form only a small part of the syllabus for other health professional students.

The majority of the participants in the survey were female. This reflects the demographics of the dental interns currently admitted to the undergraduate dental program [20]. Similar patterns related to sex were noted in Indian studies by Srivastava et al. [21], and Fotedar et al., [9], as well as in countries like Malaysia [22], Nepal [23] and Brazil [24]. Internship is the 5th year of the undergraduate dental program where students work closely with patients 6 days a week in an academic year. Thus, dental interns were included in the study because they have experienced the full breadth of the dental curriculum, and their scope of practice includes exclusive clinical practice with patient interaction. The results would thus reflect the effectiveness of the present dental curriculum related to oral cancer prevention and early detection. There were no significant differences in the scores related to the questionnaire survey among sexes. These results are similar to other studies in literature [18, 22–25]. However, gender difference in the findings will need to be explored with appropriate sampling technique depending on the demographic patterns.

Majority of the cohort had good scores related to knowledge and practice for prevention and early diagnosis of oral cancer, however, the number of individuals with good scores related to attitude was relatively less. Good knowledge among students reflects the importance given to the topic of oral cancer in the undergraduate curriculum. Knowledge is the foundation required to develop the necessary skills and attitude needed among the health professionals to provide quality healthcare services. It should be noted that skills of communication, clinical examination and clinical reasoning along with knowledge are integral to clinical outcomes; the assessment of which is beyond the scope of this survey design. Dental interns currently practice according to the expected standards of the academic institution. They have rotational postings in community as well as hospital based clinical environments, with mandated supervised clinical procedural requirements, including the practice of screening for oral cancer. As it is a mandated activity, students would be more compliant to take up such initiatives, which could in turn contribute to the good practice scores. Attitudes can greatly influence the behaviours of health professionals which directly influence patient care. Approximately 50% of the cohort included in this study had unfavourable attitudes towards prevention and early detection of oral cancer. Literature shows varied results related to attitude scores, with 74.2% and 81.4% of participants having acceptable attitude score in the study by Chan et. al, [22], and Shadid et.at [25] respectively. In contrast, the study by Shubayr et.al. [18] had 83.6% of its participants with poor scores. It is well known that approximately 90% of oral cancer cases are associated with modifiable risk factors like tobacco and alcohol [26]. Thus, the endeavours of prevention of oral cancer largely involves risk modification that are linked to lifestyle. According to literature, the likelihood of a health professional executing a lifestyle modification intervention is influenced by several factors. These factors include, commitment related to practising intervention, client receptiveness, role responsibility congruence and scope to make a difference [27]. Habit counselling for tobacco and alcohol can be challenging endeavour for health professionals as the cessation rates and patient responses are poor [28]. Additionally, dental health professionals may find it difficult to deal with psychological and social components of patients that need to be addressed for successful habit cessation behaviour. All these challenges could negatively influence the commitment of dental health professionals to engage in risk factor modifications intervention. Additionally, perceived capacity to bring about change in patient behaviour can affect clinical practice decisions of health professionals [28]. We believe that interprofessional collaboration can improve attitudes of health professionals in dealing with complex health problems including oral cancer prevention.

There are several studies that have examined the knowledge, attitude and practice of dental students related to oral cancer. A majority of the participants in the study by Srivastava et.al,[21], Gaddikeri et al. [29], and Hamdy et al. [30], reported that students lacked sufficient knowledge regarding the prevention and early detection of oral cancer (97.14%, 63.4%, 81%, and 80%, respectively). Shubayr et al. [18], noted an overall poor score for knowledge (71.9%), attitude (83.6%) and practice (62.9%) related to oral cancer prevention. Shadid et al. [25], in a similar study, reported that majority of the participants had poor knowledge and skills related to oral cancer prevention. The contrasting variability of the results in the extant literature compared to results in the present study, could possibly reflect the inclusion criteria of these studies. Along with dental interns these studies have included third- and fourth-year students, unlike our study which included only dental interns. As dental interns have more experience in comparison to their academic juniors, the knowledge of this cohort would be better. Compared to few other studies [29, 30], our questionnaire focused exclusively on prevention and early detection of oral cancer and did not include components related to advanced stages of oral cancer. Additionally, the questionnaire was developed considering an interprofessional group of health care related specialties that can contribute to prevention and early detection of oral cancer, whereas the above studies aimed to develop a questionnaire focused on dental students. It should be noted that previous literature [9, 21] and the present study indicate willingness of the participants to learn more about oral cancer prevention, indicating a need to expand curriculum or provide additional continuing education programs related to oral cancer prevention.

In the present study, most students (69.2%) had a good overall score for RIPLS with mean scores comparable to studies conducted by Salazar et al., [31] Morison et al. [32], and Mohammed et al. [33], among dental students. There is an increasing call to adopt the principles of interprofessional collaboration and the introduction of IPE courses is the foundation for future interprofessional practice. IPE courses have noted the changes in attitudes and behaviors of students with increasing tendency to adopt teamwork in a health care setting [33]. The results of the RIPLS score can be used as a baseline during implementation of future IPE courses and can help measure the outcomes of such courses. Thus, the behaviors and skills required for successful interprofessional collaboration for prevention and early detection of oral cancer can be strengthened through introduction of a focused IPE course. Also, the results indicate that favorable scores of KAP related to prevention and early detection of oral cancer have high odds of having good RIPLS score, although it was not statistically significant. It can also be noted that the highest odds of having good RIPLS score was noted in relation to good knowledge score, followed by good practice score and good attitude score. Additionally, the study findings also indicate that the students having good scores related to knowledge, practice and attitude domains of oral cancer prevention and early detection have a higher probability of having good RIPLS scores. Those with unfavorable scores of knowledge and practice had an equal probability of having good RIPLS score. Additionally, it should be noted that those with unfavourable attitude scores had a higher probability of having good RIPLS score in comparison to those with unfavourable knowledge or practice score. Poor attitudes scores may reflect the difficulty faced by dental students in influencing behavior change. These limitations may influence individuals to be open to collaboration to overcome challenges and thus the students with poor attitude scores may have a higher probability of having good RIPLS scores. Males also had a higher probability of higher scores for RIPLS, although there was no statistically significant difference between genders. A previous study in Sweden that examined gender differences in RIPLS score showed that females were more likely to have a positive attitude towards teamwork [34]. The variation in the results of this study could be attributed to lower sample of males included, and the variation in profession of the examined students. Readiness of the students related to IPE, and their willingness to learn from other professionals as well as share their knowledge with other health professionals is crucial to good outcomes related to IPE [35]. The study results indicate that students are willing to participate in an IPE module and are also interested in participating in additional training programs related to prevention and early detection of oral cancer. Dental students with poor scores would need additional resources to bridge the gap prior to participating in IPE course and such learning activities should be planned accordingly. The willingness of most of the students to learn about oral cancer prevention, and about habit cessation indicates a need to evaluate the present curriculum and to consider providing additional courses potentially related to interprofessional collaboration. The findings also indicate strengthening curricular content and assessment strategies that ensure student learning related to health promotion communication.

The results show that the students can benefit from an interprofessional course on oral cancer prevention and early detection. However, there are a few limitations related to the study that need to be considered. The results of this study represent the findings from two dental colleges in south India, with non-probability sampling techniques and needs to be generalized with caution. These findings can also benefit from qualitative perspectives to gain deeper understanding, especially related to attitudes of dental students related to oral cancer prevention and early detection and gender differences in probability of RIPLS scores. The higher proportion of females is another limitation of the study. Although, it signifies a representative sample for the Indian context, studies exploring gender differences will need to be consider sampling techniques to include a more balanced gender distribution. Future studies are planned to include other health professionals and conduct qualitative explorations.

## Conclusion

The objectives of this study were to establish a tool to assess the knowledge attitude and practice (KAP) related to prevention and early detection of oral cancer of health professionals, and to assess the same KAP of pre-licensure dental students. We also explored the possibility that these students would demonstrate good scores related to early detection and prevention of oral cancer thus indicating their readiness for interprofessional collaborative learning.

In relation to its objectives, this study reveals good scores related to knowledge and practice related to prevention and early detection of oral cancer. However, the attitude scores were lower in a relatively higher proportion of participants and needed to be addressed in the curriculum. The RIPLS score indicates a positive attitude towards interprofessional learning. Better scores related to prevention and early detection of oral cancer increase the probability of positive attitudes to interprofessional learning. The results also provide good baseline information for planning future IPE courses for prevention and early detection of oral cancer.

## Supplementary Information

Supplementary Material 1.

## Data Availability

The datasets generated and/or analysed during the current study are not publicly available due institutional policy but are available from the corresponding author on reasonable request.
